# Compression Bioreactor-Based Mechanical Loading Induces Mobilization of Human Bone Marrow-Derived Mesenchymal Stromal Cells into Collagen Scaffolds In Vitro

**DOI:** 10.3390/ijms21218249

**Published:** 2020-11-04

**Authors:** Carolina Gamez, Barbara Schneider-Wald, Karen Bieback, Andy Schuette, Sylvia Büttner, Mathias Hafner, Norbert Gretz, Markus L. Schwarz

**Affiliations:** 1 Section for Experimental Orthopaedics and Trauma Surgery, Orthopaedics and Trauma Surgery Centre, Medical Faculty Mannheim, Heidelberg University, 68167 Mannheim, Germany; Carolina.Gamez@medma.uni-heidelberg.de (C.G.); Baerbel.Schneider6@gmx.de (B.S.-W.); as.work@aikq.net (A.S.); 2 Institute of Transfusion Medicine and Immunology, Medical Faculty Mannheim, Heidelberg University, German Red Cross Blood Service Baden Württemberg—Hessen, 68167 Mannheim, Germany; Karen.Bieback@medma.uni-heidelberg.de; 3 Department for Statistical Analysis, Faculty of Medicine Mannheim, Heidelberg University, 68167 Mannheim, Germany; Sylvia.Buettner@medma.uni-heidelberg.de; 4 Institute of Molecular and Cell Biology, Mannheim University of Applied Sciences, 68163 Mannheim, Germany; m.hafner@hs-mannheim.de; 5 Institute of Medical Technology, Heidelberg University & Mannheim University of Applied Sciences, 68163 Mannheim, Germany; 6 Medical Research Centre, Medical Faculty Mannheim, Heidelberg University, 68167 Mannheim, Germany; Norbert.Gretz@medma.uni-heidelberg.de

**Keywords:** articular cartilage regeneration, compression bioreactor, mesenchymal stromal cells, MSCs, collagen scaffolds, alginate-laminin scaffolds, cell mobilization, intermittent mechanical stimulation, mechanical loading

## Abstract

Articular cartilage (AC) is an avascular tissue composed of scattered chondrocytes embedded in a dense extracellular matrix, in which nourishment takes place via the synovial fluid at the surface. AC has a limited intrinsic healing capacity, and thus mainly surgical techniques have been used to relieve pain and improve function. Approaches to promote regeneration remain challenging. The microfracture (MF) approach targets the bone marrow (BM) as a source of factors and progenitor cells to heal chondral defects in situ by opening small holes in the subchondral bone. However, the original function of AC is not obtained yet. We hypothesize that mechanical stimulation can mobilize mesenchymal stromal cells (MSCs) from BM reservoirs upon MF of the subchondral bone. Thus, the aim of this study was to compare the counts of mobilized human BM-MSCs (hBM-MSCs) in alginate-laminin (alginate-Ln) or collagen-I (col-I) scaffolds upon intermittent mechanical loading. The mechanical set up within an established bioreactor consisted of 10% strain, 0.3 Hz, breaks of 10 s every 180 cycles for 24 h. Contrary to previous findings using porcine MSCs, no significant cell count was found for hBM-MSCs into alginate-Ln scaffolds upon mechanical stimulation (8 ± 5 viable cells/mm^3^ for loaded and 4 ± 2 viable cells/mm^3^ for unloaded alginate-Ln scaffolds). However, intermittent mechanical stimulation induced the mobilization of hBM-MSCs into col-I scaffolds 10-fold compared to the unloaded col-I controls (245 ± 42 viable cells/mm^3^ vs. 22 ± 6 viable cells/mm^3^, respectively; *p*-value < 0.0001). Cells that mobilized into the scaffolds by mechanical loading did not show morphological changes. This study confirmed that hBM-MSCs can be mobilized in vitro from a reservoir toward col-I but not alginate-Ln scaffolds upon intermittent mechanical loading, against gravity.

## 1. Introduction

### 1.1. Therapeutic Approaches for Chondral Defects

Articular cartilage (AC) is the hyaline tissue covering the diarthrodial joints and contributes to load distribution during locomotion [1]. It is formed by scattered chondrocytes embedded in an extracellular matrix (ECM). Collagens are the most abundant ECM component and they are mainly responsible of the tissue volume and AC mechanical properties [2]. Blood supply, nerves and lymphatic vessels are absent in this tissue [3], this is why AC has a limited intrinsic healing capacity. Chondral injuries require early and proper treatment to avoid a major degeneration of the joints [4]. The current therapeutic options for AC repair are invasive: for instance mosaicplasty, whereby several osteochondral plugs are taken from a non-degraded part of the joint and grafted in the injured region [5] or matrix-induced autologous chondrocytes implantation (MACI) that requires two surgeries, first for chondrocytes isolation and second for implantation [6].

Microfracture (MF) is a one-step procedure preferred for the majority of small chondral defects [7,8] that aims at bone-marrow (BM) stimulation by subchondral bone micro-perforations that release blood clots containing growth factors that attract progenitor cells [9]. Even when successful in reducing pain and improving function, thus contributing to repair, the MF treatment still fails to produce regeneration and a hyaline-like tissue of similar biology and mechanical composition as the natural cartilage [10].

### 1.2. Matrix-Augmented Bone Marrow Stimulation

MF of the subchondral lamella is a reliable treatment of cartilage defects inducing an in situ regeneration process [9]. It is also named “Matrix-augmented Bone Marrow Stimulation” (MABMS) when the defect is filled with a scaffold for securing the containment of mesenchymal stem/stromal cells (MSCs) from the blood clot to enhance AC regeneration [11]. Erggelet et al., demonstrated that the treatment of microfractured full thickness cartilage defects with a cell-free poly-glycolic acid scaffold promoted defect repair tissue superior to the treatment of microfracturing alone in a sheep model [12]. Furthermore, Kramer et al. showed that MSCs were “sucked” into a matrix made of collagen-I/III by covering microfractured defects of human knees [13].

Different scaffold materials seem to differ in their performance to support chondrogenic differentiation of MSCs. Collagen-II (col-II) have better chondrogenic potential than collagen-I (col-I) scaffolds but simultaneously, it also shows arthritogenic potency [14]. Therefore, the use of col-I implants is considered safer; it is widely used and clinically approved [14].

In addition, the attractiveness of the scaffold for the recruitment of MSCs could be enhanced by the use of cytokines [15] and adhesion molecules such as laminins (Ln) [16]. In particular, Ln521 located at stem cell niches has been used in monolayers and matrices to provide a robust substratum for stem cells [17]. We recently reported that functionalizing alginate scaffolds by Ln521 enhanced the MSCs mobilization into the scaffolds [18].

### 1.3. Biomechanics in the Knee Joint and the Bioreactor

Mechanical stimulation is a key factor to understand the AC regeneration in situ. The AC in the knee joint is exposed to different acting forces as compression, tension, hydrostatic pressure and shear stress [19]. For instance, strain in healthy joints oscillates between 0 to 10% for normal daily activities with peaks in the range of 7 to 23% on the tibio-femoral contact area during walking [20]. As indicated previously, MABMS promoted mobilization of cells into a scaffold. We hypothesize that mechanical stimulation plays an important role in mobilizing MSCs. Within a proof-of-concept study with a novel bioreactor system, we in fact recently demonstrated mobilization of porcine BM-MSCs (pBM-MSCs) into functionalized alginate-Ln scaffolds [18]. Our compression bioreactor is a technological device that allows applying mechanical parameters such as frequency, strain, duration and loading on scaffolds, while resembling physicochemical environment of the AC in vitro [18]. Loading is produced by direct compression as it resembles the mechanical stress applied by the opposite joint component [21]. Such loading is produced by the strain, which is determined by the change in percentage of the tissue or scaffold height [22]. Mechanical stimulation provided by bioreactors in a dynamic-fashion simulates better the physiological loading pattern in the knee joints, and is shown to boost ECM synthesis and to support transport of nutrients through the avascular AC [23,24]. Resting times with unloaded periods naturally occur during the dynamic mechanical stimulation [25], which makes intermittent dynamic loading regimes important for AC research [18,26,27,28]. As mentioned before, the right choice of scaffolds has to be taken.

Nachtsheim et al. reported a colonization of cell-free col-I scaffolds by chondrocytes originally provided in a layer located beneath the cell free scaffold [29]. However, our preliminary results suggested that mechanical stimulation mobilizes MSCs only when working together with functionalized scaffolds [18].

### 1.4. Migration and Cell Morphology

It is well known that cyclic changes in the cell shape occur during migration in a myriad of biological processes from embryogenesis, wound healing to cancer spreading, and also responding to internal and external factors as mechano-, chemo-, thermo- and electro-taxis [30]. The cells have the ability to sense external mechanical forces as compression, tension and shear stress, transforming them into biochemical signals that alter ECM gene and protein expression to maintain the tissue homeostasis or leading to pathological changes in the tissues [31]. The interaction between cells and the surrounding substrate generates changes in cell morphology [30,32,33]. By morphometric analyses, cell shapes can be quantified based on 3D features such as sphericity, volume, convex volume and surface area [32]. Thus, the analysis of cell morphology changes by given experimental conditions may give some hints for active cell migration.

### 1.5. Focus of the Study

MF considers endogenous mobilization of stem cells to the defect site in the joint. To generate an in vitro model, which could support this idea and help to optimize therapeutic strategies like MABMS, we used here a compression bioreactor to provide mechanical stimulation on a scaffold and a cell reservoir. This should allow us to study in vitro the potential factors involved in the regeneration of AC in situ [18]. Our preliminary results suggest that mechanical stimulation could induce the mobilization of pBM-MSCs into functionalized alginate-laminin (alginate-Ln) scaffolds [18]. Thus, this time we asked whether the cell recruitment of human cells is dependent on the material of the scaffold. The aim of this study was to compare the counts of mobilized human BM-MSCs (hBM-MSCs) in alginate-Ln or col-I scaffolds upon intermittent mechanical loading.

## 2. Results

### 2.1. Cells Found in the Scaffolds after Mechanical Stimulation

**Figure 1:**
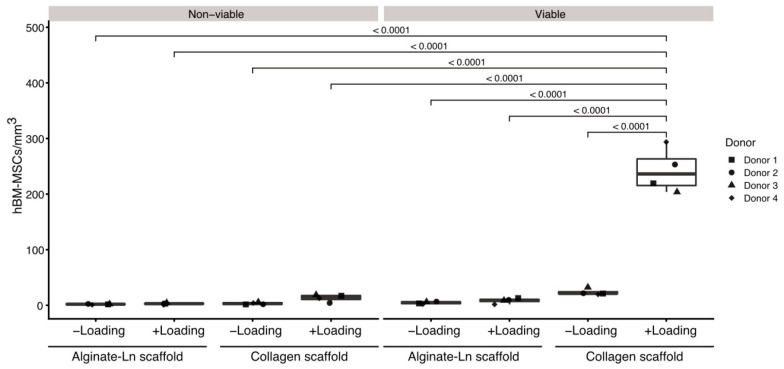
Comparison of cell counts per condition in alginate-Ln and col-I scaffolds. Mechanical loading, cell viability and type of the scaffold were statistically compared with respect to their cell counts. Col-I scaffolds contained more viable cells than alginate-Ln scaffolds. Unloaded conditions did not show significant differences in the amount of cells, independently of the viability or biomaterial of the scaffold. Examinations were done using cells from four different donors; every dot represents the mean of technical replicates. *p*-vales were estimated by a pairwise least square means comparison, and adjusted for multiple comparisons using Sidak’s correction.

**Figure 2:**
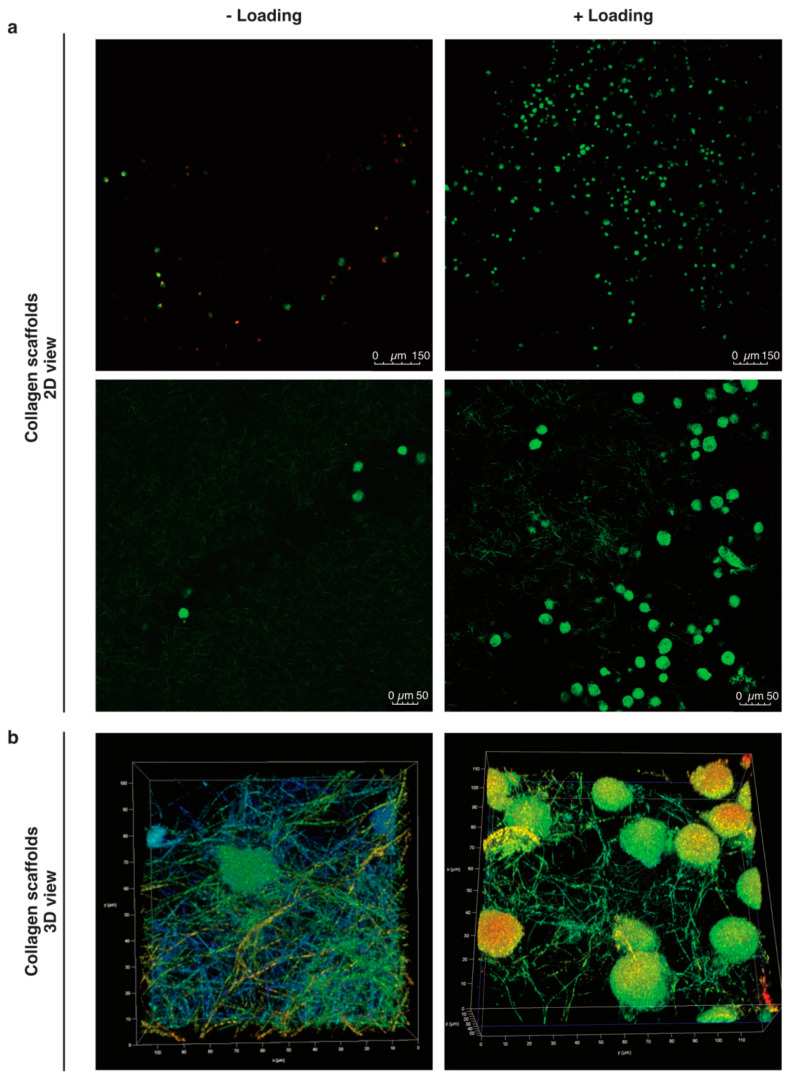
Mobilized cells into col-I scaffolds after mechanical stimulation. (**a**) The cells in the scaffolds were visualized by confocal microscopy after 24 h with or without mechanical stimulation. Viable cells are stained with C-AM (calcein-A, green) and non-viable with EthD-1 (Ethidium homodimer-1, red). (**b**) 3D images in 20× magnification plus 5X digital zoom of cells in loaded and unloaded col-I scaffolds. Pseudo-colors are used in (**b**) for a better visualization of cells within the collagen network.

Our preliminary work suggested higher counts of pBM-MSCs found in functionalized alginate-Ln scaffolds after applying intermittent mechanical loading [18]. Surprisingly, mechanical loading failed to induce mobilization of hBM-MSCs into alginate-Ln scaffolds, probably because the pore size of alginate scaffolds is too small for larger cells as hBM-MSCs [34,35,36]. We observed that hBM-MSCs were larger than pBM-MSCs under standard culture conditions (Supplementary Materials Figure S1). Thus, we chose to test col-I, a biomaterial frequently used and clinically approved for orthopedic procedures [14]. We found higher cell counts in col-I scaffolds compared to alginate-Ln scaffolds (Figure 1 and Figure 2). Using confocal microscopy, most of the cells in the scaffold were found in the adjacent side facing the cell reservoir. Figure 2 shows the distribution, cell shape, and the structure of the col-I scaffolds at different magnifications.

Loaded col-I scaffolds showed a significantly higher numbers of cells (245 ± 42 viable cells/mm^3^) in comparison to the col-I unloaded controls (22 ± 6 viable cells/mm^3^; *p*-value < 0.0001, Figure 1, Table S1); whereas a mean of 8 ± 5 viable cells/mm^3^ were found in the loaded and 4 ± 2 viable cells/mm^3^ in the unloaded alginate-Ln scaffolds (Figure 1, Table S1). Thus, mechanical loading mobilized 10-fold more cells when using col-I scaffolds in comparison with their unloaded col-I counterpart, while no significant change in the cell count was found for alginate-Ln scaffolds (Figure 1).

Based on our previous findings, we confirmed that intermittent mechanical stimulation maintains high the cell viability. For col-I scaffolds, the cell viability was 93.5% upon loading and 89.4% in the unloaded control, and 78.5% vs. 77.8% respectively for alginate-Ln scaffolds (Figure 1, Table S1); indicating that the mechanical loading did not harm the cells.

An analysis of variance (ANOVA) comparing the mean number of cell/mm^3^ for all the evaluated conditions showed that all of the experimental variables (i.e., scaffold type named as “scaffold”, loading, viability, and donor), as well as the interactions between them (scaffold *x* loading, scaffold *x* viability, scaffold *x* loading *x* viability, scaffold *x* donor, loading *x* donor, viability *x* donor, scaffold *x* loading *x* donor, scaffold *x* viability *x* donor, and scaffold *x* loading *x* viability *x* donor), had a significant effect (*p* < 0.001) on the number of cells found in the scaffolds (Tables S2–S4). However, the variable “donor” had no effect (*p* = 0.5681) and their interactions with the other variables were non-significant (*p* > 0.05). In addition, the pairwise comparison of the cell count by donor indicated that the cells from different donors used in this study behaved similarly regarding the number of cells per mm^3^ (Tables S5 and S6).

To discriminate which parameter explained the differences in the number of cells in detail, a pairwise least square means comparison was done between the counts of cells per mm^3^ for all the experimental conditions (Tables S7 and S8). We found more viable cells in col-I than in alginate-Ln scaffolds when mechanical loading was applied (*p* < 0.0001). Thus, our results show that hBM-MSCs were mobilized into col-I scaffolds upon mechanical stimulation while preserving their vitality. Figure 1 reveals the higher number of mobilized viable cells into the col-I scaffolds after loading compared with all other groups.

### 2.2. Biomechanical Stimulation Provided by the Compression Bioreactor

**Figure 3:**
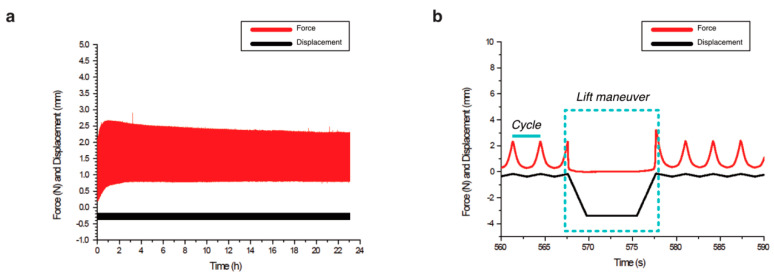
Biomechanical stimulation provided by the compression bioreactor. (**a**) Overview of a complete examination with the displacements of the piston seen in black and the resulting force in red. (**b**) Lift maneuver, i.e., the unloaded phase as part of the intermittent dynamic mechanical loading is shown in detail. Dynamic loading of repetitive cycles that compress and decompress the scaffolds was applied by the piston for 10 min. Then, an unloaded phase is reached (lift maneuver), in which the piston releases the scaffold for 10 s.

We applied intermittent dynamic mechanical stimulation as seen in detail in Figure 3. The mechanical parameters of piston displacements, number of periods, total time for the examinations and the resulting forces were analyzed (Table 1).

We found that the executed mechanical stimulation was similar for col-I and alginate-Ln scaffolds. The displacements of the piston were 202.20 ± 11.10 µm for col-I scaffolds and 277.90 ± 53.01 µm for alginate-Ln (Table 1). This higher displacement (27% approximately) found in alginate-Ln scaffolds was in the range of the normal values of strain in healthy joints [20]. The reacting force, resulting from the piston displacements (Figure 3) was 1.16 ± 0.42 N for the col-I scaffolds and 1.08 ± 0.13 N for alginate-Ln scaffolds (Table 1). A peak force was observed in every period at the end of the lift maneuver (Figure 3b), corresponding to a faster displacement of the piston when restarting the dynamic mechanical stimulation.

### 2.3. Morphometry of Mobilized Cells into Col-I Scaffolds

**Figure 4:**
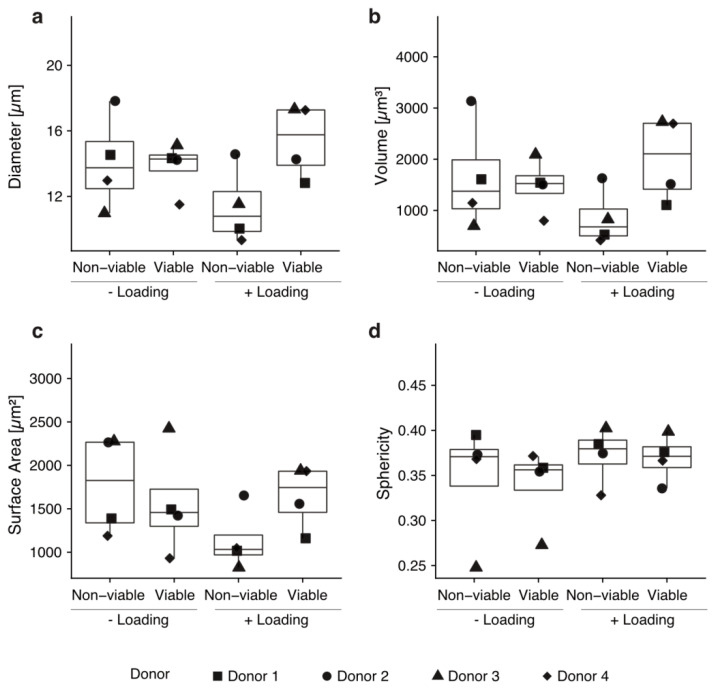
Cells that mobilized into the scaffolds by mechanical loading show no morphological changes. No statistically significant morphological changes were observed regarding diameter (**a**), volume (**b**), surface area (**c**) or sphericity (**d**) by performing a Wilcoxon rank sum test. Every condition was tested with technical triplicates of hBM-MSCs from four different donors.

We hypothesized that active migration upon mechanical loading should go in line with morphological changes of the cells. Thus, we performed morphometric analyses on the cells found in the col-I scaffolds. We addressed diameter, volume, surface area and sphericity of the cells [37]. No significant differences in cell morphology were found (*p* > 0.05, Figure 4). The sphericity of viable cells was 0.339 ± 0.045 for unloaded scaffolds and 0.369 ± 0.026 for loaded scaffolds, indicating a non-spherical shape in all cases and no significant changes between the conditions (Figure 4).

## 3. Discussion

We hypothesize that mechanical loading could help to promote regeneration in situ by mobilizing stromal cells into a scaffold placed in an AC defect. Particularly, mechanical stimulation may induce cell migration from the bone marrow reservoir to a scaffold placed in the AC defect, resembling a situation of cells moving toward the tibia plateau when the subchondral bone is opened. Within our previous work, we developed a bioreactor system as a tool to study in vitro the phenomenon of cell mobilization under controlled biomechanical load patterns like frequency and strain [18]. In that preceding proof-of-concept study, we showed that pBM-MSCs can be mobilized toward functionalized scaffolds against gravity. Now, we addressed whether human cells, specifically hBM-MSCs, could be efficiently mobilized into alginate-Ln or col-I scaffolds in this set-up. Surprisingly, we did not find many hBM-MSCs in loaded alginate-Ln scaffolds. We argue that it happened because hBM-MSCs are more elongated than pBM-MSCs (Figure S1) [36], pore size is critical and does not allow the larger human cells to enter the scaffold. Indeed, using col-I-scaffolds high cell numbers were found. In fact, cell numbers were comparable between pBM-MSCs in loaded alginate-Ln scaffolds (194 cells/mm^3^) [18] and hBM-MSCs in loaded col-I scaffolds (245 cells/mm^3^). Nevertheless, different pore sizes in alginate beads have been obtained depending on alginate or crosslinker concentration, and temperature of polymerization [38]. Such parameters could be modified (e.g., using diluted alginate solution) when fabricating alginate scaffolds in order to produce scaffolds of bigger pore sizes and therefore, it is likely that the human cells could mobilize in. As our previous work suggested that alginate-Ln worked for porcine cells, we consider that alginate-Ln might still be a suitable biomaterial if scaffolds with greater pores are produced. Thus, upon adapting the scaffold to the respective cell of interest, the used bioreactor system is helpful in optimizing strategies to mobilize endogenous stromal cells into scaffolds, which may contribute to understanding in situ regeneration.

### 3.1. Mechanical Stimulation and Mobilized Cells

Mechanical stimulation has been described as an inducing factor of non-hypertrophic chondrogenic differentiation in studies using MSCs [10]. For instance, intermittent mechanical stimulation has shown to promote expression of chondrogenic markers of BM-MSCs in alginate composites [39].

In our bioreactor, the mechanical stimulation was induced by displacements of the piston that were set at the beginning of the experiments and then monitored by an independently working sensor [18]. We observed that the set values of piston displacements (i.e., 200 µm, corresponding to 10% strain of the 2 mm of scaffold height) slightly varied from the actual executed displacements, ranging from 10.1% (col-I scaffold) to 13% (alginate-Ln scaffolds) in the present study. However, these values were in the range of diurnal to post-activity physiological strain measurements according to Sanchez-Adams et al. [20].

In line with our results of mechanical stimulation helping to recruit cells, Nachtsheim et al., found colonization of chondrocytes in an upper located col-I scaffold when using unconfined compression [29]. Nevertheless, in our system the scaffolds were stressed in a confined situation as we placed a circular compressible ring around the scaffold [18]. In our opinion, the confined compression better resembles the clinical situation of MF and MABMS than the unconfined compression. Specifically, before drilling the bone, a stable cartilage rim (i.e., the cartilage wall around the AC defect) is required to protect the blood clot or to hold the scaffold, respectively, against shear stress and destruction [11,40]. Thus, the cartilage surrounding the defect was simulated by an elastic ring made of silicone that recreated a laterally confined environment for the scaffold in our in vitro system [18]. Nachtsheim et al. detected fewer chondrocytes (approximately 150 viable cells/mm^3^, 90% cell viability) in the scaffolds after 20 days of stimulation [29] than we did after 24 h for col-I scaffolds (245 cells/mm^3^, 93.5% viability), probably because long runs may imply a higher risk of losing cells by apoptosis and necrosis. In the present study, the viability of the moved cells was slightly higher under dynamic stimulation than the viability of the controls without mechanical stimulation, which is comparable with the results of Nachtsheim et al. [29]. The higher percentage of cell viability found in loaded scaffolds may relate to an improved distribution of nutrients and better gas exchange, as the cell culture medium located on top might be more accessible for the cells upon intermittent loading.

In contrast to our results, Ode et al. reported that the mobilization of MSCs was impeded by loading in another bioreactor system [41,42]. The pneumatic load transmission used by them may exert a different load transmission pattern than our bioreactor system, which works with a step motor, moving a stiff piston that pushes directly on the scaffold and the surrounding silicone ring [18,41,42]. In addition, the mechanical stimulation patterns of Ode et al. differed from ours in terms of frequency, strain, duration and intermittent load application (i.e., they used 1 Hz and 20% strain for 72 h, whereas we applied 0.3 Hz and 10% strain for 24 h) [18].

### 3.2. Migration of Cells

The interaction between cells and the surrounding substrate generates changes in cell morphology. Stiffer substrates allow lamellipodial or mesenchymal migration, i.e., adherence-dependent migration, by the formation of focal adhesions, cell elongation and increased cell membrane area; whereas softer substrates facilitate non-adherence migration and the cells appear more rounded [30,32,33]. We argued that a change in cell morphology would allow us to discriminate active from passive mobilization of the cells into the scaffolds. Unfortunately, we were not able to calculate any significant change or trends of morphological changes indicative of cytoskeleton rearrangement after 24 h (Figure 4). Hence, it remains a matter for future studies to unravel how the cells got from the cell reservoir upward the col-I scaffold after mechanical stimulation. In an in vivo study with a sheep model, acellular implants were found to harbor functional cells after MF [12], but the mechanism of cell enrichment in the scaffolds still remains unclear. Our previous results suggested that mechanical stimulation enhanced the fluid to move upward to the scaffold [18], indicating that the cells might be passively sucked rather than actively migrated. Although if the cells were mobilized only by mechanical actions from the bioreactor, a similar ratio between viable and non-viable cells between loaded and unloaded observations should be expected, but more viable cells were found in the loaded scaffolds. Furthermore, if microfluidics given by the mechanical stimulation was the only cause of moving the cells upward, we would have expected to see a homogenous distribution of cells along the scaffold. However, the cells were mainly found in the neighboring side of the cell reservoir, probably because we had col-I scaffolds with heterogeneous pores (Figure 2), which in 3D might act as steric obstacles when the cells are passively moved upward. On the other hand, we observed a less compactness of the collagen meshwork of loaded scaffolds (Figure 2), possibly because mechanical loading had an impact on the porosity of the col-I material, encouraging the cells to mobilize. Thus, we could speculate regarding a combined system, probably moving the cells into in the scaffolds, but also promoting the cells to migrate.

Evidence supporting our findings regarding mobilized cells against gravity are cancer cells that polymerize actin to form protrusions [43] and activate their migration pathways as MMP-2, MMP-9, under microgravity environments [44]. Given the fact that pBM-MSCs required Ln functionalization [18], we argue that the cells actively displace mainly by lamellipodial migration interacting with the substrate via focal adhesions and integrins [45]. Collagen fibers may serve as lanes for integrin anchoring for migration of hBM-MSCs [46]. Thus, we consider that lamellipodial migration could be assessed in future studies by knockdown assays or inhibitors of molecules of the RhoA-ROCK-Myosin-II pathway to elucidate whether actomyosin contractility is modified when mechanical loading is applied.

### 3.3. Col-I Scaffolds

Collagens are prominent in the ECM, with col-II being the main component in AC [47]. Nevertheless, implants made of col-II do not have clinical approval yet [14], whereas col-I scaffolds or composites thereof of different shapes and sizes have been produced in the past for several applications [48,49,50], showing promising outcomes for regenerative therapies for AC defects [14,51,52,53]. The network architecture of the collagen determines its mechanical properties [54], and thus a successful polymerization is of high relevance. BDDGE as chemical compound has been used in the past at low concentrations for crosslinking hyaluronic acid, concentrated collagen solutions or collagen composites [49,50,55]. We were successful in polymerizing the col-I scaffolds with BDDGE (as described in the methods section) that were suitable for living cells and able to withstand the applied mechanical loading (Figure 2 and Figure S2).

### 3.4. Relevance and Drawbacks of Our Study

In summary, in this study we showed that: (1) the compression bioreactor was able to provide mechanical stimulation on different types of scaffolds, (2) intermittent loading induced the mobilization of hBM-MSCs from a cell reservoir toward a scaffold located above, (3) more hBM-MSCs were found upon loading when using col-I scaffolds, and (4) the cells found in the scaffolds remained viable after mechanical stimulation. Still, this study raised several questions to be addressed in further studies. Higher col-I solution concentration, possibly resulting in stiffer scaffolds could allow for even better migration [56], and longer stimulation times could potentially influence the viability of the cells or further increase the count of cells in the scaffolds. Supplementing the scaffolds with cytokines or growth factors may increase mobilized cell numbers [57], and higher cell numbers within the scaffold are needed to evaluate the stemness state of the cells after loading and to induce chondrogenic differentiation markers like SOX-9, Col-II, aggrecans and glucosaminoglycans.

We uphold the idea that a holistic perspective should be addressed to understand the healing of a complex tissue as the AC. This in vitro study showed enriched acellular col-I scaffolds with hBM-MSCs against gravity by biomechanical means. We consider that the presented bioreactor is a useful tool to examine variables in vitro possibly acting on diarthrodial joints. Thus, we hope that this study may contribute to the common efforts of improving and understanding AC regeneration in situ.

## 4. Materials and Methods

### 4.1. Cells

hBM-MSCs (experimentation approval No. 2015-520N-M, Institute of Transfusion Medicine and Immunology, Medical Faculty Mannheim of the University of Heidelberg) were isolated, expanded and characterized as described previously [58]. The cells were cultured with low glucose Dulbecco’s Modified Eagle’s Medium (DMEM, D5546, Sigma, Schnelldorf, Germany), supplemented with Fetal Bovine Serum (FBS, Cat. No. F9665, Sigma), 200 mM L-Glutamine (Cat. No. BE17-605E, Lonza, 1X Penicillin-Streptomycin (A8943, 0100, AppliChem, Darmstadt, Germany). 1× phosphate buffered saline (PBS, Cat. No. BE17-516F, Lonza, Cologne, Germany) and 0.25% Trypsin-EDTA 1× (Cat. No. 25200-056, Gibco) were used for washes and passaging. 1 × 10^3^ cells/cm^2^ were seeded in flasks for subcultivation. Passages 2–4 were used for the experiments. For the mechanical evaluation in the bioreactor, 1 × 10^5^ cells were plated in the cell reservoir.

### 4.2. Fabrication of the Scaffolds

Lyophilized sodium alginate (Keltone LVCR, ISP, USA) was mixed with 0.9% NaCl under constant stirring until dissolve to obtain 1.5% alginate solution. The obtained viscous solution was carefully sifted in a 0.22 μm filter and human Ln521 (Cat. No. LN521, Biolamina; Stockholm, Sweden) was added to a final concentration of 15 µM. The solution was then laden in holes of a custom-made sterile mold and the alginate polymerization was induced by 0.1 M CaCl_2_ for 7 min. Scaffolds with the dimensions of 10 mm diameter and 2 mm height were unmold and washed in 1X PBS.

Col-I scaffolds were made from bovine collagen solution (3 mg/mL, Cat. No. C4243, Sigma-Aldrich, Schnelldorf, Germany) and cross-linked by 1,4-Butanediol diglycidyl ether (BDDGE, Cat. No. 220892, Sigma), used at 10% according to Shankar et al. [59]. The polymerization of the gel was obtained at room temperature (RT) with pH 5.0 for 48 h.

### 4.3. Biomechanical Stimulation

The bioreactor system used in this study has previously been described in detail [18]. Briefly, it consists on a mechanical device for simultaneous loading application and cell cultivation, and a computational station for launching the setup and recording the mechanical data. The loads are exerted directly on a scaffold by a piston located on top that compresses it when displaces. A separate sensor is used for an independent measuring of the displacements. A cell reservoir is located below the scaffold; thus, the load is transmitted through the scaffold. A force sensor that measures the loads is located beneath the cell reservoir, hence, the force results of the piston displacements on the scaffold. The bioreactor is an integral system that allowed applying a mechanical setup with a robust recording data 50 Hz, i.e., the mechanical parameters as piston displacement with the resulting force and time were recorded at a rate of 50 data per second, while executing during the whole run.

To evaluate whether mechanical stimulation could induce the mobilization of the cells from the cell reservoir compartment toward the scaffold, two identical units of cell cultivation (named as “cartridge”; details previously published in [18]) were prepared, each containing 1 × 10^5^ hBM-MSCs located at the cell reservoir and the respective scaffold was placed on top. To prevent sedimentation of cells at the bottom of the cell reservoir, the cells were seeded in a solution of supplemented-DMEM and non-polymerized 0.5% col-I. Then, 2 mL of supplemented-DMEM were added onto the scaffold. For every examination, one of the cartridges was placed in the bioreactor for mechanical stimulation, while the other cartridge was kept under the same experimental conditions, but no loading was applied, serving as the unloaded control. We analyzed the mechanical data of the runs executed in the bioreactor and the number of mobilized cells within the alginate-Ln or col-I scaffolds along their cell viability. The mechanical stimulation setup was applied as previously established [18]. In short, it consisted of runs of 24 h applied in a dynamic fashion; the scaffolds were loaded at 0.3 Hz frequency, i.e., the piston displaced about 200 µm to compress the scaffold with 10% strain with respect to their initial height (2 mm) and then decompress it, repetitively for 0.3 s per cycle. This dynamic loading regime was applied intermittently, i.e., the dynamic loading took place until an unload phase of 10 s was reached, every 180 cycles; it is called here “lift” maneuver because the piston completely released the scaffold surface. Above the scaffold, cell culture medium was placed to replenish the cells with nutrients and also to avoid gas-liquid microturbulences during the lift maneuvers.

We used a confined system, in which the scaffold was held in place laterally by the elastic ring made of silicone that surrounded it [18]. During the lift maneuvers, the piston completely released the scaffold surface; thus, a permeable membrane with a mesh pore size of 160 µm was anchored above the scaffold to avoid being displaced. Preventing any disturbance between the gas atmosphere and the liquid during the lift maneuver, the piston was submerged in culture medium during the whole examination [18].

Cells from four different donors evaluated in triplicates were used for the examinations. The donors of the examinations for alginate-Ln and col-I scaffolds were the same. As controls for mechanical stimulation, cells and scaffolds were prepared in parallel and treated under the same conditions within identical cartridges as the testing samples, but no loading was applied.

### 4.4. Mechanical Data Analysis

Biomechanical data such as piston displacement, force produced by the applied periodical displacement, frequency, cycles and real duration of every examination were recorded at 50 Hz for the whole duration of every examination. The recorded data were processed and analyzed using the Origin software. Only values corresponding to dynamic mechanical stimulation were taken into account for the descriptive statistics analysis of force and piston displacement, i.e., for values such as lift maneuvers ±2 cycles, the first and the last cycles were removed for every examination. For the statistical analysis of number of cycles and total time of the tests, the complete data of the examination were considered.

Cells from four different donors were used for the examinations; the examination of every donor was tested in triplicates. For one of the replicates in col-I scaffolds was not possible to read out the mechanical data. Thus, that replicate was excluded from the statistical evaluation.

### 4.5. Confocal Microscopy

Confocal laser scanning microscopy (TSP8, Leica Microsystems, Wetzlar, Germany) was used to visualize the scaffolds and to assess the number and viability of the mobilized cells. The viability of cells within the scaffolds was assessed using calcein-AM (C-AM) to stain viable cells an ethidium homodimer-1 (EthD-1) to stain dead cells [60], using the Live/Dead kit (Cat. No. L3224, ThermoFisher, Waltham, MA, USA). After 24 h of intermittent dynamic load (or unload for controls), the cells were stained by immersing the scaffolds in a solution of 0.9% NaCl with 7 µM C-AM (Ex 494 nm/Em 517 nm) and 5 µM EthD-1 (Ex 528 nm/Em 617 nm).

The quantification of the cells inside the scaffolds was done using Leica Application Suite X (LAS X) from Leica Microsystems. For this, the 10× magnification immersion objective and green (Alexa 488 for C-AM) and red channels (Texas red for Eth-D1) were used to visualize the viable and non-viable cells, respectively. DMEM without red phenol (Cat. No. 11570406, Thermo Fisher Sci. GmbH, Dreieich, Germany) was used as immersion media when using 10× immersion objective and type F immersion oil (Cat. No. 1513859, Leica Microsystems, Wetzlar, Germany) when 20× objective. Image acquisition was done with a resolution of 2048 × 2048 pixels in all cases at 600 Hz and bidirectional scanning [18].

Col-I scaffold structure was stained using 1:200 col-I antibody (COL1A, Cat. No. sc59772, Santa Cruz Biotechnology Inc., Heidelberg, Germany) overnight at 4 °C and 1:400 IgG Alexa Fluor 488 (Cat. No. A28175, Thermo Fischer Scientific, Waltham, MA, USA) for 1 h at room temperature. Col-I was visualized and imaged by confocal microscopy using a 63× objective with type F immersion oil as immersion medium.

### 4.6. Cell Quantification in 3D and Cell Morphometric Evaluation

Detection and quantification of the cell number in the scaffolds was done by image analysis using LAS X software using a validated pipeline [18,37]. Briefly, the software GUI allowed the creation of a custom pipeline for image analysis based on algorithms. The background noise reduction was obtained by 3D median filtering and the discrimination between cells and background was obtained using the adjust threshold option. Binary data were then processed using morphological filters such as 3D whole filling and opening filter with radius sphere of 2–3 voxels. Based on the shape and intensity, morphometric features as volume, sphericity, surface area and diameter were calculated by LAS X. The objects with sphericity equal to 1 were considered completely spherical [61]. A range of 8–25 µm was selected for analysis, assuming that cells were within that range as previously reported [62,63].

As previously mentioned, C-AM and EthD-1 allowed us to discriminate between the viable and non-viable cells within the scaffolds, respectively. Thus, the counts of cells were normalized in the volume of the imaged region of interest (i.e., 1.1 mm width × 1.1 mm length × 2.0 mm height). The mean of the viable and non-viable cells per mm^3^ of the four donors was used to determine the percentage of cell viability for every condition.

### 4.7. Statistical Analyses

Descriptive statistical analyses were performed using Origin version 9.0G for Windows (OriginLab Corporation, Northampton, MA, USA). The box plots were made in R Core Team 2019 (Foundation for Statistical Computing, Vienna, Austria) using the data obtained from Origin and SAS software.

Analysis of variance (ANOVA) was performed using SAS 9.4 (Business Analytics und Business Intelligence Software, Cary, NC, USA). The General Linear Model (GLM) procedure was used to fit general linear model, obtaining a *p*-value for the whole model as an indicator of a significant effect for at least one of the variables. The tested model was composed of the variables mechanical stimulation (loading or no loading), cell viability, type of scaffold and donor. To discriminate within the variables causing a significant change in the model, a pairwise least square means comparison was performed comparing between the counts of cells per mm^3^ among the factors and their levels all the experimental conditions. *p*-values were calculated in a paired fashion. The model was adjusted for multiple comparisons using the method of Sidak [64].

For all cases, *p*-values lower than 0.05 were taken as statistically significant.

## 5. Conclusions

Our results show that intermittent mechanical stimulation induces the mobilization of hBM-MSCs toward col-I scaffolds counter gravity, but not toward alginate-Ln scaffolds as used in the present study. We found that the cell count in col-I was statistically significant compared to the unloaded control and the alginate-Ln scaffold. In addition, the majority of the mobilized cells are viable, which is of high relevance for the future research aiming chondrogenic differentiation. With this study, we can confirm that hBM-MSCs can be mobilized into a scaffold when using intermittent mechanical stimulation along with an appropriated scaffold biomaterial as col-I. The results in vitro shown here may contribute to the better understanding of human AC regeneration in situ.

## 6. Patents

An application for a Model Utility Protection for the bioreactor (No. 2019 103 387.8) has been filed on 17 June 2019.

## Figures and Tables

**Table 1:** Descriptive statistics of the biomechanical stimulation applied on the scaffolds. Several parameters as displacement of the piston, force, time and number of periods were analyzed for the examinations. n: number of examinations executed in the bioreactor.

Scaffold	Data	n	Mean	Std Dev	Median	Max	Min
Alginate-Ln	Force (N)	12	1.16	0.42	1.15	2.23	0.69
	Displacement (µm)	12	277.90	53.01	287.93	359.01	174.05
	Number of periods	12	142.58	16.42	151.50	156.00	108.00
	Time (h)	12	24.13	0.08	24.16	24.21	23.95
Col-I	Force (N)	11 *	1.08	0.13	1.09	1.25	0.88
	Displacement (µm)	11 *	202.20	11.10	199.68	225.73	193.18
	Number of periods	11 *	133.27	10.73	141.00	143.00	118.00
	Time (h)	11 *	23.64	0.86	23.40	24.95	22.52

* One of the technical replicates was excluded (see Section 4.4).

## Supplementary Materials

Supplementary Materials can be found at https://www.mdpi.com/1422-0067/21/21/8249/s1.

## Abbreviations

AC	Articular cartilage
Alginate-Ln	Alginate-Laminin
BM	Bone marrow
BDDGE	1,4-Butanediol diglycidyl ether
C-AM	Calcein-AM
Col-I	Collagen type-I
Col-II	Collagen type-II
ECM	Extracellular matrix
EthD-1	Ethidium homodimer-1
GLM	General linear model
hBM-MSCs	Human bone marrow derived mesenchymal stromal cells
LAS X	Leica Application Suite X
Ln	Laminin
pBM-MSCs	Porcine bone marrow derived mesenchymal stromal cells
MABMS	Matrix-augmented bone marrow stimulation
MACI	Matrix-augmented chondrocytes implantation
MF	Microfracture
MSCs	Mesenchymal stem/stromal cells
