# Supplementary material for: Compression Bioreactor-Based Mechanical Loading Induces Mobilization of Human Bone Marrow-Derived Mesenchymal Stromal Cells into Collagen Scaffolds In Vitro
Source: Int J Mol Sci. 2020 Nov 4;21(21):8249. doi: 10.3390/ijms21218249 (PMC7672606; doi:10.3390/ijms21218249)
Supplement: Supplementary file 1 [file ijms-21-08249-s001.pdf]

Supplementary tables

Table S1. Descriptive statistics of mobilized hBM-MSCs into alginate-Ln and col-I scaffolds. Quantification of viable/dead cells present within alginate-Ln and col-I scaffolds with/without mechanical loading. The mechanical stimulation did not affect the cell viability. n= 4, number of donors from which technical replicates were performed.

Scaffold	Group	Viability	Mean (cells/mm^3)	Std Dev	Median	Max	Min
Alginate-Ln	-Loading	Non-viable	1	1	1	2	0
	-Loading	Viable	4	2	4	6	1
	+Loading	Non-viable	2	2	2	4	0
	+Loading	Viable	8	5	9	12	1
Col-I	-Loading	Non-viable	3	2	3	5	1
	-Loading	Viable	22	6	21	32	17
	+Loading	Non-viable	17	12	17	32	3
	+Loading	Viable	245	42	237	300	205

Analysis of variance (ANOVA) comparing the model

The SAS software was used to perform an **Analysis of variance (**ANOVA) of the model to compare the model composed by the variables: type of scaffold ("scaffold"), mechanical stimulation ("loading"), cell viability ("viability") and donor ("donor"). The GLM (Generalized Linear Model) procedure was applied for weighted analysis with "Cells per mm^3" as dependent variable.

The GLM Procedure
Dependent Variable: Cells per mm^3
Weight: weighting

Table S2. ANOVA table. The overall *F* test is significant (F = 53.16 and $p < 0.001$), indicating that the model as a whole accounts for a significant amount of the variation in "cells per mm^3". This shows that it is appropriate to test the effects.

Source	DF	Sum of Squares	Mean Square	F Value	Pr > F
Model	31	201267.9784	6492.5154	53.16	<.0001
Error	60	7328.0256	122.1338		
Corrected Total	91	208596.0040			

DF: degrees of freedom, MSE: mean square error, SS: sum of squares.

Table S3. Simple Statistics. The R-Square indicates that the model accounts for 96 % of variation in "cells per mm^3"**.** The coefficient of variation, Root Mean Square for Error, and mean of the dependent variable are also listed.

R-Square	Coeff Var	Root MSE	Cells per mm^3 Mean
0.964870	29.84819	11.05141	37.02540

MSE: mean square error, Coeff Var: coefficient of variation.

Table S4. Test of effects of type III sum of squares. According to a significance level of 5 %, the interaction between donor with any of the other variables (scaffold, loading, or viability) is not significant ($p > 0.05$ in all cases between the interactions with donor). This indicates that donor does not depend of the variables scaffold, loading, nor viability, and vice versa. There is a significant effect ($p < 0.05$) for the individual variables scaffold, loading, and viability, and their respective interactions, but not for donor ($p < 0.05$). Looking at the interaction between the variables Donor, Scaffold, Loading and Viability, $p = 0.0388$ indicates a difference in cell count.

Source	DF	Type III SS	Mean Square	F Value	Pr > F
Scaffold	1	35649.81445	35649.81445	291.89	<.0001
Loading	1	27555.15549	27555.15549	225.61	<.0001
Viability	1	33674.21779	33674.21779	275.72	<.0001
Donor	3	248.91832	82.97277	0.68	0.5681
Scaffold *x* Loading	1	25426.44509	25426.44509	208.19	<.0001
Scaffold *x* Viability	1	29532.38296	29532.38296	241.80	<.0001
Scaffold *x* Loading *x* Viability	2	44246.28733	22123.14366	181.14	<.0001
Scaffold *x* Donor	3	634.93416	211.64472	1.73	0.1699
Loading *x* Donor	3	558.30072	186.10026	1.52	0.2176
Viability *x* Donor	3	531.87300	177.29100	1.45	0.2368
Scaffold *x* Loading *x* Donor	3	756.08832	252.02944	2.06	0.1146
Scaffold *x* Viability *x* Donor	3	701.87587	233.95862	1.92	0.1367
Scaffold *x* Loading *x* Viability *x* Donor	6	1751.68482	291.94747	2.39	0.0388

DF: degrees of freedom, SS: sum of squares.

Statistical pairwise comparison of the cell count by donor

Pairwise least square means comparison between the counts of cells per mm^3 for the donors. The calculations were made using the SAS software.

ANOVA: Comparisons (Alginate-Ln/Col-I scaffolds, Donor)

——————————————————

The GLM Procedure
Least Squares Means
Adjustment for Multiple Comparisons: Sidak

Table S5. Least Square-Means of "Cells per mm^3" for every donor.

Donor	Cells per mm^3 LSMEAN	LSMEAN Number
Donor 1	34.5022383	1
Donor 2	37.3481749	2
Donor 3	34.7921832	3
Donor 4	41.4590220	4

LSMEAN: Least squares means.

Least Squares Means for effect Donor
Pr > |t| for H0: LSMean(i)=LSMean(j)
Dependent Variable: Cells per mm^3

Table S6. p-values for pairwise LS-Mean matrix comparing the differences of the cell count regarding the donor. The multiple comparison analysis show that the different donors have similar effects regarding the dependent variable "Cells per mm^3".

i/j	1	2	3	4
**1**		0.9964	1.0000	0.7622
**2**	0.9964		0.9980	0.9752
**3**	1.0000	0.9980		0.7954
**4**	0.7622	0.9752	0.7954	

Statistical pairwise comparison of the cell count in alginate-Ln and col-I scaffolds

Pairwise least square means comparison between the counts of cells per mm^3 for all the experimental conditions. The calculations were made using the SAS software.
ANOVA: Comparison (Alginate-Ln/Col-I scaffolds)
____

The GLM Procedure
Least Squares Means
Adjustment for Multiple Comparisons: Sidak

Table S7. Least Square-Means of "Cells per mm^3" for the conditions "scaffold", "loading" and "viability".

Scaffold	Loading	Viability	Cells per mm^3 LSMEAN	LSMEAN Number
Alginate-Ln	+ Loading	Non-viable	2.066667	1
	+ Loading	Viable	7.541667	2
	- Loading	Non-viable	1.108333	3
	- Loading	Viable	3.875000	4
Col-I	+ Loading	Non-viable	12.534435	5
	+ Loading	Viable	243.336777	6
	- Loading	Non-viable	2.634298	7
	- Loading	Viable	23.106061	8

LSMEAN: Least squares means.

Least Squares Means for effect Scaffold*Loading*Viability
Pr > |t| for H0: LSMean(i)=LSMean(j)
Dependent Variable: Cells per mm^3

Table S8. p-values for pairwise LS-Mean matrix comparing the differences of the cell count for the conditions "scaffold", "loading" and "viability". The multiple comparison analysis show that the condition with "col-I scaffold", "+ loading" and "viable cells" had significant effects regarding the dependent variable "Cells per mm^3" with respect to the other conditions.

i/j	1	2	3	4	5	6	7	8
**1**		1.0000	1.0000	1.0000	0.9993	<.0001	1.0000	0.3791
**2**	1.0000		1.0000	1.0000	1.0000	<.0001	1.0000	0.8869
**3**	1.0000	1.0000		1.0000	0.9972	<.0001	1.0000	0.2993
**4**	1.0000	1.0000	1.0000		1.0000	<.0001	1.0000	0.5553
**5**	0.9993	1.0000	0.9972	1.0000		<.0001	0.9997	0.9992
**6**	<.0001	<.0001	<.0001	<.0001	<.0001		<.0001	<.0001
**7**	1.0000	1.0000	1.0000	1.0000	0.9997	<.0001		0.4315
**8**	0.3791	0.8869	0.2993	0.5553	0.9992	<.0001	0.4315	

Supplementary figures

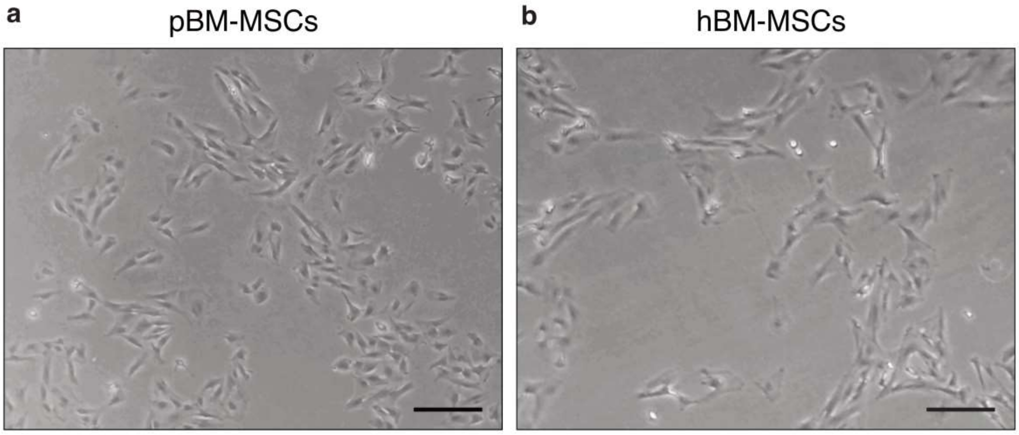

Figure S1. Comparison of porcine and human cell sizes in culture. pBM-MSCs **(a)** are smaller than hBM-MSCs **(b).** Both images show cells in passage 1. *Scale bar: 100 µm.*

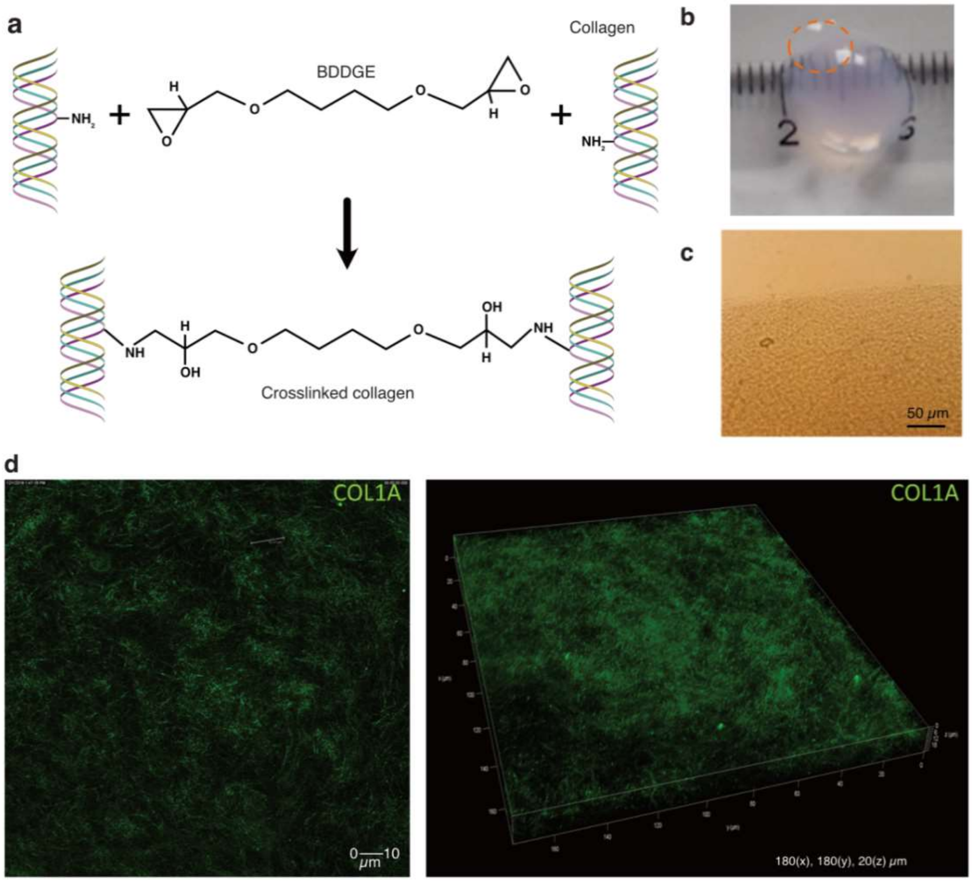

Figure S2. Preparation of col-I scaffolds. The col-I scaffolds were prepared using 10 % BDDGE as crosslinker of col-I solution at pH 5.0 and room temperature for 48 hours. **(a)** Schematic representation of the crosslink between collagen and BDDGE. The amine groups from the collagen react with the terminal epoxide residues of BDDGE, causing the crosslink between collagen molecules. **(b)** Stable scaffolds in shape were obtained as confirmed by the boundaries of the scaffold seen macro- (b) and microscopically (c). **(c)** Optical microscopic view of the col-I scaffolds edge (orange circle in b) at 10X. **(d)** Confocal microscopy of the col-I scaffold network with COL1A staining. Dimension of the 3D image: 180 (x), 180 (y), 20 (z) µm. *BDDGE: 1,4-Butanediol diglycidyl ether, COL1A: monoclonal antibody against collagen-1A.*
